# Effects of Menaquinone-7 on Bone Turnover Markers, Femoral Mechanical Resistance, and Histology in Young Ovariectomized Rats

**DOI:** 10.3390/nu18101510

**Published:** 2026-05-09

**Authors:** Alexandru Jecan, Gheorghe Tomoaia, Luciana-Mădălina Gherman, Vasile Rus, Raluca Maria Pop, Cătălin Popa, Răzvan Marian Melinte, Diana Jecan-Toader, Dragoș Apostu, Luca Simionescu, Vlad Blănaru, Daniel Oltean-Dan

**Affiliations:** 1 Department of Orthopedics and Traumatology, University of Medicine and Pharmacy “Iuliu Hațieganu”, 400132 Cluj-Napoca, Romania; jecan.alexandru@elearn.umfcluj.ro (A.J.); profgtomoaia.umfcluj@yahoo.com (G.T.); contact@drapostu.ro (D.A.); luca.simionescu@elearn.umfcluj.ro (L.S.); olteandandaniel@yahoo.com (D.O.-D.); 2 Orthopedics and Traumatology Clinic “Alexandru Radulescu”, Emergency County Hospital, 400347 Cluj-Napoca, Romania; def.vlad@gmail.com; 3 Experimental Centre, University of Medicine and Pharmacy “Iuliu Hațieganu”, 400349 Cluj-Napoca, Romania; luciana.gherman@umfcluj.ro; 4 Department of Histology, Faculty of Veterinary Medicine, University of Agricultural Sciences and Veterinary Medicine Cluj-Napoca, Calea Mănăștur No. 3-5, 400372 Cluj-Napoca, Romania; vasile.rus@usamvcluj.ro; 5 Department of Pharmacology, Toxicology and Clinical Pharmacology, University of Medicine and Pharmacy “Iuliu Hațieganu”, 400012 Cluj-Napoca, Romania; raluca_parlog@yahoo.com; 6 Department of Materials Science and Engineering, Technical University of Cluj-Napoca, 103-105, Muncii Ave., 400641 Cluj-Napoca, Romania; catalin.popa@stm.utcluj.ro; 7 EUt+ Institute of Nanomaterials & Nanotechnologies EUTINN, European University of Technology, European Union; 8 Department 2, Faculty of Nursing and Health Sciences, University of Medicine and Pharmacy “Iuliu Hațieganu”, 400132 Cluj-Napoca, Romania; dtoader28@gmail.com; 9 2nd Pediatric Clinic, Emergency Clinical Hospital for Children, 400124 Cluj-Napoca, Romania

**Keywords:** osteoporosis, vitamin K2, menaquinone-7, MK-7, young ovariectomized rat model, bone mechanical resistance, bone turnover markers, osteocalcin

## Abstract

Background: Osteoporosis is a major skeletal disorder, particularly affecting postmenopausal women. Young ovariectomized rat models are commonly used to investigate estrogen deficiency-related skeletal changes, although they do not fully reproduce osteoporosis in a mature postmenopausal skeleton. Established pharmacological therapies remain the cornerstone of osteoporosis management, while nutritional factors continue to be investigated for their potential supportive role in bone metabolism. Menaquinone-7 (MK-7), a form of vitamin K2, has been investigated for potential skeletal effects through vitamin k-dependent mechanisms, particularly osteocalcin carboxylation. The aim of this study was to evaluate the dose-dependent effects of MK-7 on bone turnover markers, femoral mechanical resistance, qualitative histological findings, and hepatic safety in a young ovariectomized rat model. Methods: Forty female Wistar rats that were 8 weeks old, and thus still undergoing skeletal maturation, were assigned to four groups: sham-operated controls, ovariectomized controls, ovariectomized rats treated with low-dose MK-7, and ovariectomized rats treated with high-dose MK-7. Treatment was administered every 48 h for 12 weeks. At study completion, 35 rats survived; standardized analysis included eight animals per group. Plasma bone turnover markers (BTMs) and alanine aminotransferase were measured, femoral strength was assessed by the three-point bending test, and bone and liver histology was analyzed. Results: Biomechanical testing showed that high-dose MK-7 was associated with greater femoral mechanical resistance compared with untreated ovariectomized rats, while qualitative histology suggested differences in cortical architecture among groups. Biochemically, MK-7 treatment reduced undercarboxylated osteocalcin, suggesting vitamin K-dependent target engagement, whereas conventional turnover markers showed discordant findings. Overall, hepatic architecture was preserved, although mild hepatocellular apoptosis was observed. Conclusions: In this young OVX rat model, high-dose MK-7 was associated with improved femoral mechanical resistance compared with untreated OVX controls. However, because ovariectomy was performed during skeletal maturation, these findings should be interpreted as preliminary and cannot be directly extrapolated to established postmenopausal osteoporosis in a mature skeleton, and further studies are needed to clarify its activity pathways and safety profile.

## 1. Introduction

Osteoporosis is the most prevalent systemic skeletal disorder, characterized by low bone mass, microarchitectural deterioration, and increased bone fragility. It is the leading cause of fragility fractures, predominantly affecting postmenopausal women [1]. With the rapid aging of the global population, osteoporosis has escalated into a critical public health challenge.

In the European Union alone, the annual economic burden exceeds €56.9 billion, a cost attributed solely to the management of fractures and associated long-term disabilities [2]. Similarly, data from the United States indicate a significant treatment gap: among 2.3 million osteoporotic fractures in a single year, only 9% of patients received appropriate screening and treatment [3].

Established pharmacological therapies remain the cornerstone of osteoporosis management and have demonstrated efficacy in reducing fracture risk. In parallel, nutritional factors continue to be investigated for their potential supportive role in bone metabolism, particularly in relation to bone turnover, matrix protein activation, and mineral homeostasis. Calcium and vitamin D are established nutritional components of bone health, although they are not substitutes for antiosteoporotic pharmacotherapy when drug treatment is indicated. Within this broader nutritional context, vitamin K2 has attracted interest because of its role in the gamma carboxylation of vitamin K-dependent proteins involved in bone metabolism [3,4]. Vitamin K2 (menaquinone) has emerged as a promising option for ameliorating bone health. Increased intake of MK-7 through high consumption of natto in the Japanese population has been associated with a reduced risk of osteoporotic fractures in postmenopausal women [5].

Unlike vitamin K1, vitamin K2, specifically the menaquinone 7 isomer (MK-7), has a superior bioavailability and bioactivity in extrahepatic tissues. Its primary mechanism involves the activation of vitamin K2-dependent proteins (VKDPs) such as Osteocalcin via gamma-carboxylation, a step essential in binding calcium to the bone matrix for proper mineralization. Furthermore, MK-7 is shown to enhance bone matrix production and increase collagen synthesis, which is an essential factor in bone health and regeneration [6]. Additionally, MK-7 plays an important role in activating Matrix Gla Protein (MGP), with potential effects on preventing vascular calcification; the dual action has contributed to interest in vitamin K-dependent pathways in both skeletal and vascular biology [7].

Assessment of potential skeletal interventions requires integration of complementary endpoints. Bone turnover markers (BTMs) provide dynamic information regarding systemic remodeling activity and may respond earlier than structural outcomes. Therefore, *C*-terminal telopeptide of type I collagen (CTXI) was used as a marker of bone resorption, while procollagen type I *N*-terminal propeptide (PINP) and bone alkaline phosphatase (BALP) were used as markers of bone formation. In addition, undercarboxylated osteocalcin (ucOC) was selected as a marker of vitamin K-dependent osteocalcin carboxylation. Because biochemical markers cannot directly define bone architecture or mechanical competence, femoral three-point bending testing and qualitative histology were also included as terminal functional and descriptive structural endpoints. Finally, alanine aminotransferase (ALT) and liver histology were assessed to explore hepatic safety [8,9,10,11].

The aim of this study was to evaluate the dose-dependent effects of MK-7 on bone turnover markers, femoral mechanical resistance, qualitative histological findings, and hepatic safety in a young ovariectomized rat model. Because ovariectomy was performed during skeletal maturation, the model should be interpreted as an estrogen-deficiency model in young rats rather than as a direct equivalent of established postmenopausal osteoporosis in a mature skeleton.

## 2. Materials and Methods

### 2.1. Ethical Statement

All animal procedures were strictly aligned with international ethical guidelines (EU directive 2010/63/EU). Ethical clearance was obtained from the Institutional Ethics Committee of the “Iuliu Hațieganu” University of Medicine and Pharmacy of Cluj Napoca (Approval No. AVZ 13/2 February 2024), as well as from the National Sanitary Veterinary and Food Safety Authority (ANSVSA) (Approval No. 397/28 February 2024).

Forty female Wistar Albino rats, 7 weeks old, weighing between 150 and 180 g, were included in the study. At this age, rats are still undergoing skeletal maturation; therefore, the model was considered a young OVX estrogen deficiency model, with potential effects on both peak bone mass acquisition and OVX induced bone remodeling.

The animals were housed in standard polycarbonate cages under strictly controlled environmental conditions (temperature 22 ± 2 °C, humidity 55 ± 5%, 12 h light/dark cycle). The animals had ad libitum access to standard rodent food (Safe D40 maintenance diet Safe diets, Augy, France) and water. To minimize stress and anxiety, animals were handled using handling tunnels and non-aversive techniques throughout the experiment.

In accordance with the 3Rs principle (Replacement, Reduction, and Refinement), femoral bones were harvested bilaterally to maximize the sample size while using the minimum number of animals. All surgical and euthanasia procedures were performed in accordance with European guidelines to minimize animal suffering and under the guidance and supervision of a veterinary surgeon.

### 2.2. Experimental Design and Surgical Procedure

Following a 7-day acclimatization period, the animals were randomly divided into four groups:

1. Sham-operated control (sham, *n* = 10): Subjected to sham surgery and treated with corn oil (Sigma-Aldrich, C8267, CAS 8001-30-7; Merck KGaA, Darmstadt, Germany) by oral gavage every 48 h.
2. Ovariectomized control (OVX, *n* = 10): Subjected to bilateral ovariectomy and treated with corn oil by oral gavage every 48 h.
3. Low-dose MK-7 (OVX LD, *n* = 10): OVX animals treated with 0.06 mg/kg body weight every 48 h (equivalent to daily dose of 0.03 mg/kg).
4. High-dose MK-7 (OVX HD, *n* = 10): OVX animals treated with 0.1 mg/kg body weight every 48 h (equivalent to daily dose of 0.05 mg/kg).

#### Surgical Procedure

All surgical interventions were performed under strict aseptic conditions by a blinded investigator, utilizing sterilized instrumentation and disposable sterile drapes. A multimodal anesthesia and analgesia protocol was implemented to ensure optimal perioperative care. Pre-operatively, subjects were weighed to ensure precise dosing. General anesthesia was induced via intraperitoneal injection of Ketamine (Bela-pharm GmbH & Co. KG, Vechta, Germany), (75 mg/kg) and medetomidine (0.5 mg/kg) (Orion corporation, Espoo, Finland) cocktail. Following confirmation of a deep surgical plane of anesthesia, subjects were placed in dorsal recumbency; the ventral abdominal region was then shaved and disinfected with a 10% povidone-iodine solution (Betadine, EGIS Pharmaceuticals PLC, Budapest, Hungary). A 5 cm midline laparotomy was performed, with meticulous hemostasis maintained through electrocautery. The intestinal loops were gently retracted laterally to achieve optimal visualization of the ovaries. In the ovariectomized (OVX) groups, the ovarian and broad ligament were carefully isolated and transected bilaterally using electrocautery to ensure definitive hemostasis, followed by the complete excision of both ovaries. In the sham-operated group, the ovaries were identified and exteriorized but remained intact, undergoing the same surgical stress without excision.

Prior to abdominal closure, the peritoneal cavity was thoroughly lavaged with pre-warmed sterile saline (0.9% NaCl) to prevent hypothermia and remove any residual debris. The surgical site was then prepared for a layered closure: the musculoaponeurotic layer of the abdominal wall was sutured using a continuous locking pattern with 2/0 Vicryl (polyglactin 910; Ethicon Inc., Somerville, NJ, USA), ensuring a secure and tension-free seal. The tegument was subsequently closed with simple interrupted sutures using 2/0 Biopro (Polypropylene; Biosintex, Cluj-Napoca, Romania). The incision site was disinfected with povidone iodine and treated with a topical oxytetracycline spray (Terramycin 3.92% Zoetis UK Limited, Leatherhead, Surrey, UK) to prevent secondary infections. Following the procedure, the animals were placed in a temperature-controlled recovery area and monitored continuously until the full restoration of the righting reflex and spontaneous locomotor activity.

### 2.3. Pharmacological Treatment

Vitamin MK-7 (Menaquinone-7, MK-7, MilliporeSigma, Sigma-Aldrich, PHR 2363, CAS 2124-57-4, Burlington, MA, USA) was administered via oral gavage every 48 h for 12 weeks, starting the day after surgery. A stock solution (1 mg/mL) was prepared by dissolving 75 mg MK-7 in 75 mL corn oil. Working solutions were prepared by mixing the stock solution with corn oil heated to 40 °C to achieve concentrations of 0.018 mg/mL (low dose) and 0.03 mg/mL (high dose). Solutions were stored at 2–8 °C. Prior to administration, solutions were gently heated to 40 °C and agitated to ensure homogeneity and redissolve any potential precipitates. Dosage volumes were adjusted weekly based on body weight, which was measured weekly. All treatments were administered by a blinded researcher.

### 2.4. Sample Collection

At the conclusion of the 12-week experimental period, 35 of the initial 40 rats survived. The animals had originally been allocated to four equal groups of 10 subjects each. Three rats died during the surgical procedure: one in the sham group, one in the OVX LD group, and one in the OVX HD. In addition, during the pharmacological treatment phase, oral gavage, particularly when repeated over a prolonged period, represents a significant stress factor, and two further deaths occurred: one in the OVX LD group and one in the OVX control group. Thus, at end of the experiment, the surviving animals were distributed as follows: sham (*n* = 9), OVX control (*n* = 9), OVX LD (*n* = 8), and OVX HD (*n* = 9). To standardize the groups for sample collection and subsequent analyses, 8 animals per group were included. To ensure a balanced design for ANOVA and subsequent group comparisons, 8 animals per group were included in the final standardized analysis. In groups with more than 8 surviving rats, each animal was assigned a number from 1 to 9, and one number was randomly selected via a computer to determine which rat would be excluded from the analysis.

Blood samples were collected from the caudal vein under light sedation to minimize animal distress. Blood collection was performed in the morning, at approximately 08:00, to reduce circadian variability. However, fasting status was not controlled, as animals had ad libitum access to food before sampling. Samples were drawn into tubes containing ethylenediaminetetraacetic acid (VACUETTE EDTA, Greiner Bio-One GmbH, Kremsmunster, Austria) as an anticoagulant. To obtain plasma, the samples were centrifuged at 2500 rpm (approximately 650 times g) for 5 min within 20 min of collection. The resulting plasma was aliquoted and stored at −80 °C until further biochemical analysis. Following blood sampling, the animals were euthanized via an anesthetic overdose, ensuring a painless and rapid termination in accordance with ethical guidelines for tissue harvesting. Immediately post-mortem, the liver and both femurs were excised. The femurs were meticulously cleaned of adherent soft tissue and fixed in 10% neutral buffered formalin for subsequent biomechanical testing and histological evaluation (n = 16 femurs, 8 allocated to histology and 8 to biomechanical testing). Similarly, liver specimens were fixed in 10% formalin for histopathological assessment of systemic toxicity.

### 2.5. Biochemical Analysis

Plasma bone turnover markers were quantified using enzyme-linked immunosorbent assay (ELISA) kits manufactured by ELK Biotechnology (Wuhan, China), according to the manufacturer’s instructions. Rat C-telopeptide of type I collagen (CTXI) was measured using a competitive inhibition ELISA (Cat. No. ELK2221) with a kit sensitivity of 49.6 pg/mL and a detection range of 156.25–10,000 pg/mL. Rat bone-specific alkaline phosphatase (BALP) was measured using a sandwich ELISA (Cat No: ELK5682) with sensitivity of 0.3 ng/mL and a 0.79–50 ng/mL range. Rat Procollagen I *N*-Terminal Propeptide (PINP) was measured using a Sandwich ELISA (Cat. No. ELK7661) with 1.18 ng/mL and a 3.13–200 ng/mL range. Rat undercarboxylated Osteocalcin (ucOC) was measured using a sandwich ELISA (Cat. No. ELK9272) with 0.058 ng/mL sensitivity and a 0.16–10 ng/mL range. Absorbance readings were obtained using an 800 TS ELISA microplate reader (Agilent Technologies INC., Santa Clara, CA, USA), and plate washing was performed using a Biotek Microplate 50 TS washer (Agilent Technologies Inc., Santa Clara, CA, USA). Liver toxicity was assessed by alanine aminotransferase (ALT), REF 11533 ( BioSystems S.A, Barcelona, Spain), measured spectrophotometrically with the automatic analyzer Applied BioSystem A15 (BioSystems S.A, Barcelona, Spain). All samples were processed by a blinded researcher.

### 2.6. Biomechanical Testing

To evaluate bone functional integrity and mechanical strength, three-point bending tests were performed using a Zwick/Roell Z005 testing machine (Zwick/Roell GmbH & Co, Ulm, Germany), with data acquisition conducted with the dedicated TestXpert II software, version 3.3.0.4258 (Zwick/Roell GmbH & Co, Ulm, Germany). Femurs were placed on two support points at the level of the metaphyseal region, with the support span length standardized at 10 mm for all specimens. A progressive load at a constant displacement rate of 1 mm/min was applied at the middle of the diaphysis. Maximum load to failure and stiffness were recorded. The obtained mechanical parameters were interpreted as measures of whole-bone mechanical behavior under the standardized testing configuration, rather than intrinsic material properties.

### 2.7. Histological Analysis

Following euthanasia, liver and femoral specimens were collected. In femoral samples, the epiphyses were resected to facilitate penetration of the fixative into the medullary canal. All samples were analyzed by a blinded researcher, and systematically sampled images were selected. Liver fragments measuring 5 mm in thickness and femoral diaphyseal specimens were fixed in 10% neutral buffered formalin for 5 days at room temperature. The extended fixation period was selected because the harvested bone fragments exceeded the thickness typically recommended for short fixation protocols. Although fixation of small bone samples may be completed within 12–24 h, penetration of fixatives is slower in mineralized tissues than in soft tissues and depends on the degree of mineralization. The femoral epiphyses were sectioned to facilitate fixative penetration into the medullary cavity. Because the primary objective of histological processing was to assess general diaphyseal wall architecture, compact bone lamellae, and resorption-like/lytic areas rather than detailed cellular morphology, complete fixation was prioritized to reduce the risk of postmortem autolysis. Bone specimens were subsequently decalcified in 5% trichloroacetic acid for 30 days after completion of fixation. All specimens were dehydrated in graded ethanol, cleared in n-butanol, and embedded in paraffin. Twenty transverse serial sections with a thickness of 5 μm were cut using a rotatory microtome (Leica RM 2125; Leica Biosystems Nussloch GmbH, Nussloch, Germany) and mounted on glass slides. Five microscopic slides, which did not have any display artifacts, were subsequently stained with Goldner’s trichrome method. All histological slides were examined using a light microscope (Olympus BX41, Tokyo, Japan), and photomicrographs were captured with an Olympus cellSens Entry 3.1 software. To ensure blinded scoring, in the microscopic investigation part, only numbers (i.e., 1.2, 1.5, 2.6, etc.) were noted on the histological slides.

### 2.8. Statistical Analysis

Data were analyzed using SPSS (Version 26, release 26.0.0.0 64-bit edition; IBM Corp, Armonk, NY, USA). The individual rat was considered the experimental unit for all statistical analyses. For biomechanical and histological analyses, one femur per rat was included in each corresponding analysis to avoid pseudoreplication. Normality was assessed using the Shapiro–Wilk test, histograms, and Q-Q plots, while homogeneity of variances was evaluated with Levene’s test. As all included variables were normally distributed, continuous data were expressed as mean ± standard deviation (SD). For comparisons between groups, one-way ANOVA was used when assumptions of normality and homogeneity of variances were met, followed by Tukey’s post hoc test. In cases of unequal variances, Welch’s ANOVA was applied, followed by Games–Howell post hoc testing. A *p*-value < 0.05 was considered statistically significant.

## 3. Results

### 3.1. Biochemical Parameters

Mean values for ALT, CTXI, ucOC, BALP, and PINP across the four study groups are presented in Table 1.

In contrast, CTXI levels differed significantly among groups (Welch’s *F* = 10.805, *p* = 0.001). Post hoc Games–Howell analysis showed that the sham group had significantly higher CTXI values than both the OVX control group (*p* = 0.001) and the OVX LD group (*p* = 0.019). No difference was observed between the sham group and OVX HD, or among the OVX, OVX LD, and OVX HD groups. Post hoc groupings are summarized in Table 1.

ucOC levels also differed significantly between groups (F = 5.922, *p* = 0.004). Tukey’s post hoc testing showed significantly higher ucOC values in the sham group compared with both the OVX LD (*p* = 0.005) and OVX HD (*p* = 0.011) groups. No statistically significant difference was noted between the sham and OVX control groups. Post hoc groupings are summarized in Table 1.

PINP values showed marked intergroup differences (*F* = 32.430, *p* < 0.001). Games–Howell post hoc analysis revealed that the OVX control had significantly higher PINP levels than the sham group (*p* < 0.001) and OVX HD (*p* = 0.001). In addition, the OVX HD had significantly higher values than the sham group (*p* = 0.005). Differences between OVX LD and the other groups were not statistically significant. Post hoc groupings are summarized in Table 1.

### 3.2. Biomechanical Testing

Three-point bending testing demonstrated significant differences in whole-bone femoral mechanical resistance among groups (*F* = 19.242, *p* < 0.001).

The sham group showed significantly higher mean maximum load to failure than both the OVX control (mean difference = 65.73, *p* < 0.001) and the OVX LD group (mean difference = 56.63, *p* = 0.001), while no significant difference was observed between the sham and OVX HD groups (mean difference = 0.08, *p* = 1.000). The OVX HD group also showed significantly greater maximum load to failure than the OVX control group (mean difference = 65.8, *p* < 0.001) and OVX LD group (mean difference = 56.70, *p* < 0.001). No significant difference was identified between the OVX control and OVX LD groups (*p* = 0.827). The biomechanical results and post hoc groupings are summarized in Table 2.

### 3.3. Histological Findings

#### 3.3.1. Bone Histology

Qualitative histological examination of femoral diaphyseal sections revealed intergroup differences in cortical wall appearance, compact bone organization, and the extent of osteolytic/resorption-like areas among the OVX control, OVX LD, and OVX HD groups.

**Figure 1:**
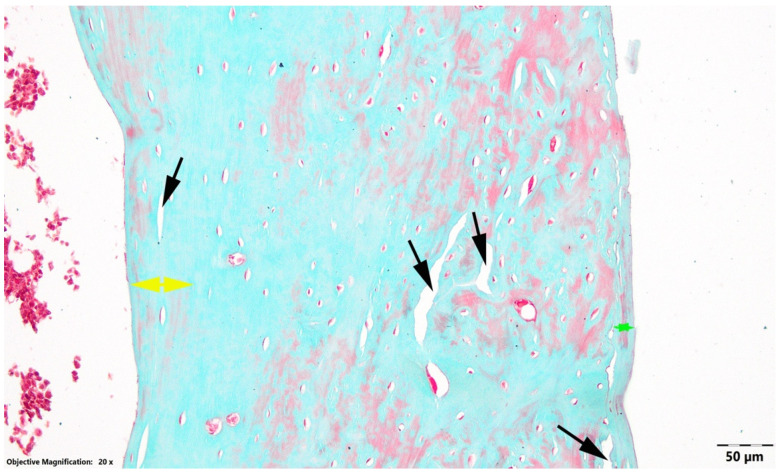
Transverse section at the diaphyseal level, OVX control group. Black arrow, areas of bone lysis; green arrow, subperiosteal compact non-Haversian bone; yellow arrow, subendosteal compact non-Haversian bone. Goldner’s trichrome stain.

In the OVX group, the diaphyseal wall was the thinnest of the three groups. The compact non-Haversian bone layers were markedly reduced in thickness, and although lytic areas did not occupy a large proportion of the sections (Figure 1), the remaining bone showed the least preserved compact cortical appearance compared with OVX LD and OVX HD.

**Figure 2:**
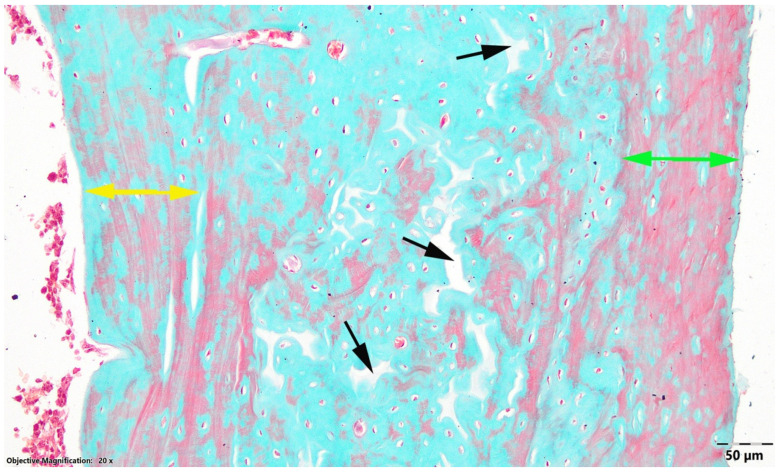
Transverse section at the diaphyseal level, OVX LD group. Black arrow, areas of bone lysis; green arrow, subperiosteal compact non-Haversian bone; yellow arrow, subendosteal compact non-Haversian bone; Goldner’s trichrome stain.

In the OVX LD group, the diaphyseal wall showed the greatest overall thickness. The subperiosteal and subendosteal compact non-Haversian bone layers were slightly thicker than in OVX HD group; however, the microscopic appearance suggested a less preserved compact cortical pattern. In addition, lytic areas were more numerous than in the OVX HD group. Areas with an osteoid-like appearance, suggestive of newly formed non-mineralized bone matrix, were also observed in both subperiosteal and subendosteal regions (Figure 2).

**Figure 3:**
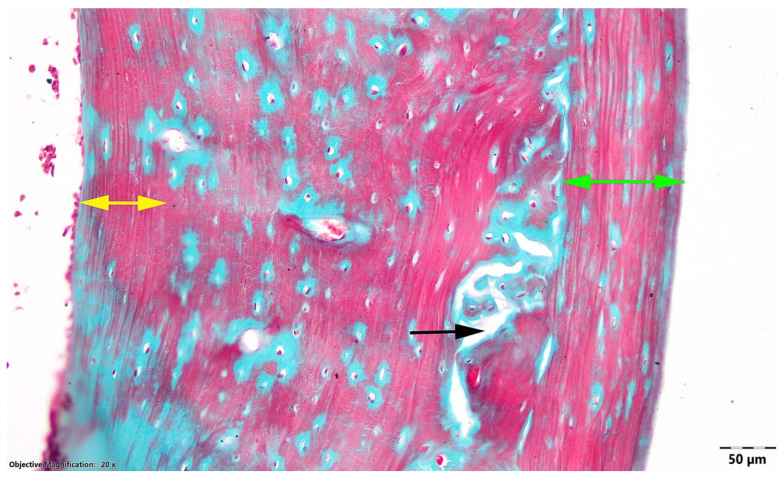
Transverse section at the diaphyseal level, OVX HD group. Black arrow, area of bone lysis; green arrow, subperiosteal compact non-Haversian bone; yellow arrow, subendosteal compact non-Haversian bone; Goldner’s trichrome stain.

In the OVX HD group, the diaphyseal wall showed the most preserved qualitative cortical architecture among the OVX groups. Both subperiosteal and subendosteal layers of compact non-Haversian bone were well represented, and no evident resorption-related lytic areas were observed in the examined sections. However, because no quantitative histomorphometry, bone mineral density, or mineral content analysis was performed, these findings should not be interpreted as direct evidence of increased bone density or mineralization (Figure 3).

Overall, qualitative histology suggested a more preserved cortical architectural pattern in the OVX HD group, intermediate changes in the OVX LD group, and the poorest cortical appearance in the OVX control group.

#### 3.3.2. Liver Histology

In both treatment groups, the overall hepatic cytoarchitecture was preserved, with no inflammatory infiltrates or major degenerative hepatocellular changes identified on routine light microscopy. The main alteration observed was hepatocellular apoptosis, with differences in both density and morphological stage between groups.

**Figure 4:**
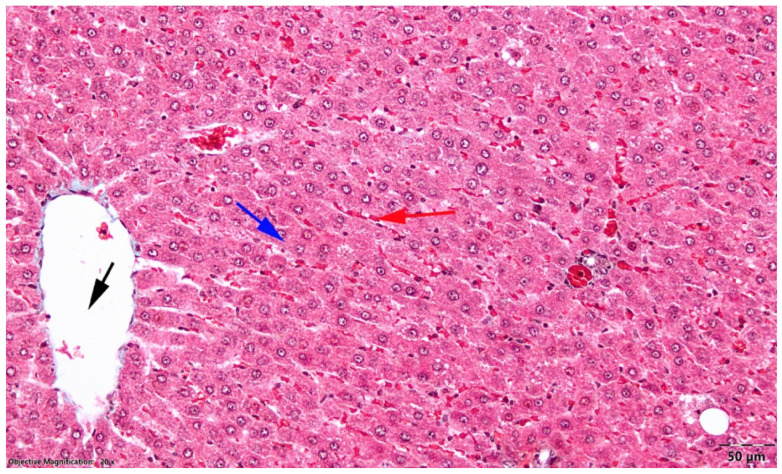
Liver, OVX control group; normal appearance of the hepatic parenchyma; black arrow, centrilobular venule; blue arrow, Remak cords; red arrow, sinusoidal capillaries; Goldner’s trichrome stain.

In the OVX control group, the hepatic cytoarchitecture appeared normal. No inflammatory infiltrates or degenerative changes of the hepatocytes were observed in the examined sections (Figure 4).

**Figure 5:**
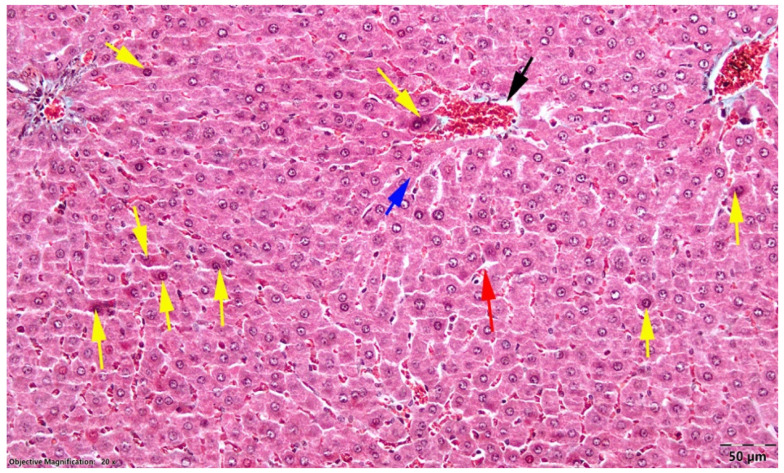
Liver, OVX LD group; moderate-to-marked hepatocellular apoptosis; black arrow, centrilobular venule; blue arrow, Remak cords; red arrow, sinusoidal capillaries; yellow arrow, apoptotic cells; Goldner’s trichrome stain.

In the OVX LD group, apoptotic hepatocytes were present in approximately 50% of examined high-power fields, but their density was generally lower, usually with fewer than five apoptotic cells per field. In addition, most apoptotic cells appeared to be in earlier stages, characterized mainly by nuclear cortical hyperchromatosis, with only rare pyknotic nuclei and less intense cytoplasmic acidophilia than in the OVX HD group (Figure 5).

**Figure 6:**
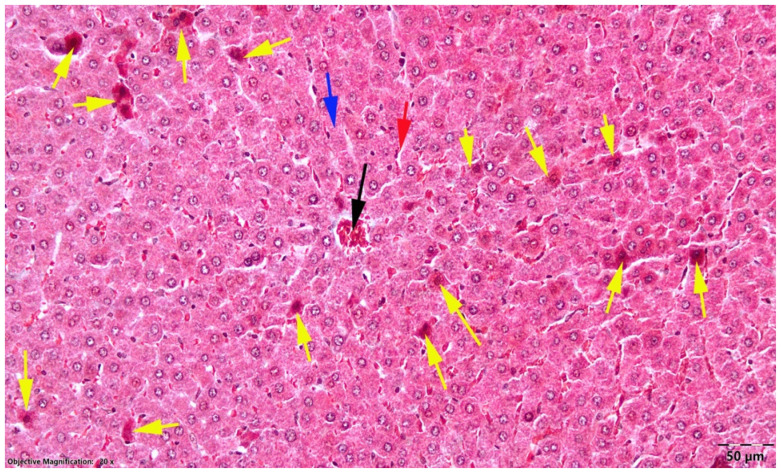
Liver, OVX HD group; relatively intense hepatocellular apoptosis; black arrow, centrilobular venule; blue arrow, Remak cords; red arrow, sinusoidal capillaries; yellow arrow, apoptotic cells; Goldner’s trichrome stain.

In OVX HD group, apoptotic hepatocytes were more numerous and more advanced morphologically. Approximately 50% of the examined high-power fields contained apoptotic hepatocytes, and in many of these fields, more than five apoptotic cells were identified. Most apoptotic hepatocytes showed advanced features, including pyknotic nuclei and occasional karyorrhexis (Figure 6).

Overall, liver histology suggested a greater degree and more advanced stage of hepatocellular apoptosis in the OVX HD than in OVX LD group.

## 4. Discussion

In this young ovariectomized (OVX) rat study, high-dose MK-7 was associated with improved femoral mechanical resistance compared with untreated OVX controls, while low-dose MK-7 showed a less consistent response. Because ovariectomy was performed at 8 weeks of age, during ongoing skeletal maturation, these findings should be interpreted in the context of estrogen deficiency affecting both peak bone mass acquisition and OVX-induced remodeling, rather than as a direct model of established postmenopausal osteoporosis in a mature skeleton. Similarly, terminal bone turnover marker values may reflect both growth-related skeletal activity and estrogen deficiency-related remodeling, rather than disease-related remodeling alone. The OVX control group showed the highest PINP values, whereas PINP was lower in the OVX HD group, which does not support a straightforward anabolic interpretation. Similarly, the CTXI pattern was atypical, with lower values in OVX controls than in sham animals and numerically higher values in the OVX HD group. Although MK-7 treatment decreased ucOC, suggesting vitamin K-dependent target engagement, the conventional bone turnover marker profile should be considered discordant and exploratory rather than mechanistically confirmatory. Hepatic histology showed preserved architecture across groups but increased hepatocellular apoptosis at high dose with unchanged ALT, suggesting a mild/subclinical hepatic affection that warrants cautious interpretation and targeted follow-up.

In the present young OVX model, high-dose MK-7 was associated with higher femoral load-bearing capacity than untreated OVX controls. However, the biological interpretation must account for the age of the animals, and whether the same mechanical and biomechanical response would occur in older skeletally mature OVX rats remains uncertain. In 8-week-old rats, estrogen deprivation may interfere not only with remodeling balance but also with growth-related skeletal development and peak bone mass acquisition. Therefore, the observed mechanical and histological differences may reflect the combined effects of ongoing skeletal maturation, OVX-induced estrogen deficiency, and MK-7 exposure. BTMs characterize dynamic whole-skeleton remodeling activity and remain relevant for interpreting treatment-related changes in bone metabolism. However, BTMs do not directly measure bone mass, mineralization, geometry, or mechanical competence. Conversely, biomechanical testing provides a terminal functional outcome, and qualitative histology provides descriptive structural information, but neither can define the underlying remodeling mechanism in the absence of BMD, mineral content analysis, micro-CT, or quantitative histomorphometry. Therefore, these endpoints should be interpreted as complementary rather than hierarchical, particularly given the discordance observed between biochemical, histological, and biomechanical findings [12,13].

Prior OVX animal studies have reported mixed effects of vitamin K2 on bone outcomes, reflecting heterogeneity in rat strain, age at ovariectomy, diet, dosing, treatment duration, and skeletal endpoints. Although we acknowledge that bone modeling and growth-related skeletal acquisition remain active at 8 weeks of age, young OVX rat models have been used in experimental studies to investigate the skeletal consequences of estrogen deficiency. At this age, ovariectomy provides an accelerated hypoestrogenic model that may be useful for studying early estrogen deficiency-related changes in bone turnover and mechanical performance. Therefore, the present model should not be considered a direct equivalent of established postmenopausal osteoporosis in a mature skeleton, but rather a young OVX estrogen-deficiency model. In this context, our findings may be relevant to the broader biological effects of hypoestrogenism on bone, including impaired peak bone mass acquisition and premature ovarian insufficiency-related bone loss. Nevertheless, extrapolation to mature postmenopausal osteoporosis should be made cautiously [13,14,15]. For example studies by Iwamoto et al. and Omelka et al. found beneficial effects of MK-7 supplementation on bone health in OVX rats [16,17]. Additional OVX literature indicates that dietary MK-7 may prevent OVX induced reductions in femoral dry weight and calcium content, potentially through its conversion to Menaquinone-4 (MK-4) in bone tissue [10]. Moreover, studies by Ikeda et al. and Kojima et al. reported that increased intake of natto, the richest dietary source of MK-7, was associated with reduced bone mass loss at the hip and distal radius, as well as lower risk of hip fractures in postmenopausal women [5,18]. In contrast, Fu et al. concluded that supplementation of phylloquinone (PK), MK-4, and MK-7 did not prevent bone loss in an OVX rat model when other nutrients, notably calcium and vitamin D, were adequate [19]. The divergence of MK-7 skeletal efficacy may depend on baseline vitamin K2 status, rat species and age, co-nutrient context (calcium/vitamin D), dosing and exposure, endpoint selection (trabecular or cortical), and the time window post-OVX [13].

International consensuses recommend plasma PINP as a reference formation marker and plasma CTXI as a reference resorption marker [9,12]. In OVX rodents, estrogen deficiency is expected to elevate both resorption and formation markers due to coupling, even though net balance becomes negative. Estrogen deficiency is associated with high levels of both resorption and formation markers and identifies PINP among commonly used formation markers in rats. Therefore, the highest PINP in the OVX control group may reflect increased formation activity coupled to estrogen deficiency-related remodeling. However, this was not paralleled by the expected CTXI pattern. Importantly, the lower PINP observed in OVX HD compared with OVX controls does not support an anabolic effect of MK-7 based on systemic formation markers. Thus, the mechanism underlying the improved mechanical results remains uncertain [11,13].

The observed decrease in ucOC in the MK-7-treated groups is a coherent pharmacodynamic signal of increased vitamin K2-dependent gamma carboxylation of osteocalcin, as percentage of ucOC is widely used as a marker of vitamin K2 status in bone [10]. Importantly, however, ucOC should be interpreted with caution. When not normalized to total osteocalcin, its interpretation may be confounded by changes in osteoblastic activity independent of vitamin K2 exposure, and clinical trials have not consistently shown that reductions in ucOC translate to decreased bone loss or fracture risk in unselected populations [10]. In this study, the reduction in ucOC therefore supports targeted engagement, indicating improved vitamin K2 status at the bone level, and is consistent with recent evidence showing that vitamin K2 supplementation reliably decreases ucOC and improves osteocalcin carboxylation, while often exerting minimal or inconsistent effect on other bone turnover markers [20].

BALP remained unchanged across groups. This finding is consistent with the emerging view that vitamin K2 interventions may improve bone health primarily through effects on osteocalcin carboxylation and matrix mineralization dynamics, rather than through marked increases in systemic bone formation marker output. In support of this interpretation, a recent meta-analysis reported significant improvements in osteocalcin carboxylation indices, including increased cOC and decreased ucOC, but no significant effects on BALP or PINP across the included trials, highlighting that osteocalcin-related changes may occur without major shifts in conventional bone-formation markers [20]. Vitamin K2 has also been shown in experimental systems to modulate osteoblast-related gene expression through nuclear receptor signaling, particularly via the SXR/PXR pathway. However, such transcriptional effects may not translate to measurable changes in circulating BALP in vivo [21]. Taken together, the unchanged BALP values and the lower PINP observed in the OVX HD group do not support a systemic anabolic marker response. Although MK-7 reduced ucOC, suggesting vitamin K-dependent target engagement, the present data do not offer a definitive biological explanation linking biochemical, histological, and mechanical findings. Therefore, the improved whole-bone mechanical resistance observed in the OVX HD group should be interpreted as a terminal functional finding with an uncertain underlying mechanism [20].

The atypical CTXI pattern should be interpreted with caution, as CTXI is among the most pre-analytically sensitive bone turnover markers. Consensus guidance indicates that CTXI is substantially influenced by circadian variation and food intake: levels peak in the early morning and may decrease by up to 40% after feeding. In the present study, blood collection was standardized to the morning, at approximately 08:00, which partially limits circadian variability. However, fasting status was not controlled, because animals had ad libitum access to food before sampling. Therefore, feeding-related variability cannot be excluded and represents an important pre-analytical limitation for CTXI interpretation. Accordingly, sampling for resorption markers should be carefully standardized and precisely timed to minimize variability. In addition, the same guidance emphasizes the importance of sample matrix and handling conditions. EDTA plasma is considered more stable than serum or heparin plasma because EDTA inhibits proteases capable of degrading CTXI epitopes. Delayed centrifugation or inappropriate sample matrices may therefore lead to misleading results, and the magnitude or even direction of change may vary according to the immunoassay used and its antibody specificity for degradation products [9]. Looking at the multiple variables involved in handling CTXI samples, pre-analytical or analytical artefacts cannot be excluded as a major contributor to the discordant findings. Additional biological explanations should also be considered. CTXI and total PINP may be influenced by renal clearance, which is an important issue in humans and may also be relevant in rodents if renal function differs between groups. Moreover, systemic bone turnover markers may not adequately capture site-specific changes in cortical diaphyseal remodeling that are more directly related to mechanical outcomes. However, because renal indices were not assessed, these explanations remain speculative in the context of this study [13]. Overall, CTXI did not provide a coherent signal in this experiment. Therefore, CTXI findings should be regarded as exploratory, and no firm conclusion regarding resorption activity can be drawn from this marker.

The present findings do not establish a single coherent biological pathway linking MK-7 exposure, systemic bone turnover markers, qualitative histology, and whole-bone mechanical resistance. The reduction in ucOC suggests vitamin K-dependent target engagement, but the conventional BTMs were discordant and did not support a clear anabolic or antiresorptive mechanism. Similarly, qualitative histology suggested differences in cortical architecture but could not establish mineralization, bone density, or histomorphometry improvement. Thus, the increased femoral mechanical resistance observed in the OVX HD group should be considered a terminal functional outcome whose biological basis remains uncertain and requires confirmation using serial BTMs, BMD, mineral content analysis, micro-CT, and quantitative histomorphometry.

Liver histology showed overall preservation of hepatic architecture. Despite unchanged ALT levels, increased hepatocellular apoptosis was observed in the treatment groups with the most hepatocellular apoptosis in OVX HD group. In the vitamin K2-safety liver literature, a 90-day rat toxicology study of synthetic MK-7 reported no compound-related toxicity at doses up to 10 mg/kg/day and established that no observed adverse effect level (NOAEL) existed at the highest tested dose, supporting a generally favorable preclinical safety profile at those exposure levels [22]. However, Fu et al., in their OVX rat model study, discussed detected MK-7 epoxide in serum and suggested that hepatic recycling capacity may be exceeded under certain dosing or dietary conditions, with uncertain long-term implications. This observation is consistent with mild apoptotic signal identified here at the highest dose [19]. Overall, the combined histological and biochemical findings suggest limited hepatic toxicity, with mild apoptotic changes that increased with the treatment dose.

There are several limitations to our study. The sample size was relatively small, which may have reduced the statistical power to detect subtle differences between groups. In addition, because ovariectomy was performed in 8-week-old rats, the model does not fully reproduce established postmenopausal osteoporosis in a mature skeleton. At this age, rats are still undergoing skeletal maturation and peak bone mass acquisition; therefore, the observed biochemical, histological, and mechanical outcomes may reflect the combined effects of estrogen deprivation, ongoing growth, and MK-7 exposure. This limits direct extrapolation to postmenopausal osteoporosis and should be considered when interpreting the results. Histologic evaluation was mainly descriptive and was not complemented by quantitative histomorphometry or micro-CT, thus limiting detailed assessment of bone microarchitecture. The histological assessment focused on the femoral cortical diaphysis, whereas OVX-induced bone loss is often more pronounced in metabolically active trabecular compartments; therefore, trabecular microarchitectural changes could not be evaluated in the present study. In addition, the biomechanical parameters obtained from three-point bending testing are influenced by bone geometry, including cross-sectional size, shape, and mass distribution. Although support span length was standardized at 10 mm for all specimens, cortical geometry, cross-sectional moment of inertia, BMD, mineral content, and calcium/phosphorus content were not assessed; therefore, it was not possible to distinguish intrinsic material effects from geometric contributions to whole-bone mechanical resistance. The biochemical analysis was also restricted to selected markers; lack of total osteocalcin measurements limits interpretation of ucOC changes, and the atypical CTXI pattern should be interpreted with caution given the known pre-analytical and biological variability of this marker and uncontrolled fasting status. Accordingly, these results should be regarded as preliminary preclinical evidence supporting a potential beneficial skeletal effect of MK-7, particularly at high doses, while highlighting the need for additional studies.

## 5. Conclusions

In this young ovariectomized (OVX) rat model, high-dose menaquinone-7 (MK-7) supplementation was associated with improved femoral mechanical resistance compared with untreated OVX controls. This finding was primarily reflected in enhanced femoral mechanical resistance, while qualitative histology suggested differences in cortical architecture. However, the discordance between biochemical markers, qualitative histology, and biomechanical findings limits mechanistic interpretation. The concomitant reduction in circulating undercarboxylated osteocalcin (ucOC) confirms effective vitamin K2 target engagement and supports the biological activity of MK-7 in promoting γ-carboxylation-dependent processes within bone tissue. In contrast, the discordant CTXI and PINP responses do not support a clear antiresorptive or anabolic mechanism. Therefore, the mechanism underlying the improved femoral mechanical resistance observed in the OVX HD group remains uncertain. When considered together, these findings suggest a possible MK-7-related effect on whole-bone mechanical performance under estrogen-deficient conditions in young rats, but they do not establish a coherent anabolic, antiresorptive, mineralization-related, or histomorphometry mechanism. However, because the animals were still undergoing skeletal maturation, the results should be considered preliminary and should not be directly extrapolated to mature postmenopausal osteoporosis. However, the observation of mild hepatic involvement warrants careful consideration and underscores the need for comprehensive safety assessments. Future studies should aim to elucidate the molecular pathways through which MK-7 exerts its skeletal effects and to further characterize its long-term safety profile.

## Figures and Tables

**Table 1:** Biochemical parameters across study groups. Data are presented as mean ± SD.

Parameter	Sham (*n* = 8)	OVX (*n* = 8)	OVX LD (*n* = 8)	OVX HD (*n* = 8)	Global *p*-Value
ALT (U/L)	79.50 ± 15.00	94.88 ± 15.25	94.63 ± 16.80	91.25 ± 20.16	0.487
CTXI (pg/mL)	1799.22 ± 103.01 ^a^	1337.19 ± 184.52 ^b^	1427.44 ± 245.66 ^b^	1722.51 ± 430.56 ^ab^	0.001
ucOC (ng/mL)	0.208 ± 0.035 ^a^	0.159 ± 0.054 ^ab^	0.110 ± 0.046 ^b^	0.118 ± 0.023 ^b^	0.004
BALP (ng/mL)	0.626 ± 0.091	0.599 ± 0.067	0.759 ± 0.213	0.600 ± 0.064	0.325
PINP (ng/mL)	2.679 ± 0.368 ^a^	10.241 ± 2.253 ^b^	7.638 ± 4.735 ^abc^	5.011 ± 1.314 ^c^	<0.001

Data are presented as mean ± SD. Values sharing at least one superscript letter are not significantly different; values with no shared superscript letters are significantly different based on post hoc testing. Tukey’s post hoc test was used for ucOC; Games–Howell post hoc test was used for CTXI and PINP. ALT and BALP did not show significant intergroup differences. No significant differences in ALT levels were observed among the groups (*F* = 0.837, *p* = 0.487). Similarly, BALP levels did not differ significantly between groups (Welch’s *F* = 1.307, *p* = 0.325).

**Table 2:** Mean values for maximum load to failure (N) in three-point bending testing.

Group	Maximum Load to Failure (N)
Sham (*n* = 8)	100.25 ± 9.81 ^a^
OVX (*n* = 8)	34.53 ± 14.45 ^b^
OVX LD (*n* = 8)	43.63 ± 28.67 ^b^
OVX HD (*n* = 8)	100.33 ± 21.78 ^a^

Data are presented as mean ± SD. Global ANOVA: F = 19.242, *p* < 0.001. Values sharing the same superscript letter are not significantly different; different superscript letters indicate statistically significant differences between groups based on Tukey’s post hoc test.

## Data Availability

The original contributions presented in this study are included in the article. Further inquiries can be directed to the corresponding author.
